# Characterization of the complete chloroplast genome of the woody sow-thistle, *Sonchus leptocephalus* (Asteraceae) endemic to the Canary Islands

**DOI:** 10.1080/23802359.2019.1674719

**Published:** 2019-10-07

**Authors:** Hye-Been Kim, Myong-Suk Cho, Seon-Hee Kim, JiYoung Yang, Seung-Chul Kim

**Affiliations:** a Department of Biological Sciences, Sungkyunkwan University, Gyeonggi-do, Republic of Korea;; b Research Institute for Dok-do and Ulleung-do Island, Kyungpook National University, Daegu, Republic of Korea

**Keywords:** The woody *Sonchus* alliance, Canary Islands, adaptive radiation, Taeckholmia

## Abstract

*Sonchus leptocephalus* belongs to a core member of the woody *Sonchus* alliance endemic to the Macaronesian Islands in the Atlantic Ocean. The alliance has been subject to intensive investigations of adaptive radiation on oceanic islands. As an attempt to fully understand the patterns and processes of evolution in this group, we determined the complete chloroplast genome of *S. leptocephalus* endemic to the Canaries. It was 152,406 bp in length, comprising 84,331 bp of large single copy and 18,583 bp of small single copy separated by 24,746 bp of inverted repeats. A total of 130 genes were determined including 87 protein-coding genes, 6 ribosomal RNA, and 37 transfer RNA genes. Phylogenetic analysis confirmed its position within the woody *Sonchus* alliance.

*Sonchus leptocephalus*, a core member of the woody *Sonchus* alliance, is a delicate shrub with small heads (15-20 florets per head) and finely dissected leaves, and grows commonly on walls, rocks and cliffs in the lower dry vegetation zones in Tenerife and Gran Canaria of the Canary archipelago (Bramwell 1997). It was originally placed in one of the segregate genera of the alliance, *Taeckholmia* (Boulos 1967); however, broadly circumscribed generic concept of *Sonchus* s.l., including *Taeckholmia* and other small and monotypic genera from the Atlantic and Pacific Islands, has gained support recently (Aldridge 1976; Greuter 2003; Kilian et al. 2009, 2009+; Mejías and Kim 2012). Of approximately 35 species of the woody *Sonchus* alliance, *S. leptocephalus* ( = *T. pinnata*) belongs to one core group having highly dissected leaves with small heads (capitula), which can be easily distinguished from other members of the alliance. Phylogenetic relationships among seven species of *Taeckholmia* sensu Boulos (1967) are poorly known with two major lineages recognized; one includes one species, *S. arboreus* ( = *T. arborea*), while the other includes the remaining species (Lee et al. 2005). Further studies are required to determine the phylogenetic relationships among them precisely, which has been an exceptional challenge due to lack of efficient molecular markers based on nuclear and chloroplast genomes. As an ongoing effort to characterize the plastid genomes of the members of the woody *Sonchus* alliance and to understand their evolutionary trends, we utilized next-generation sequencing (NGS) approach and assembled the complete chloroplast genome of *S. leptocephalus*.

Total genomic DNA of *S. leptocephalus* (voucher specimen: *SKK190602003*, SKK) was isolated from the leaves collected from Gran Canaria (28°08′31.7″N 15°35′30.5″W) using the DNeasy Plant Mini Kit (Qiagen, Hilden, Germany). NGS was performed using the Illumina HiSeq platform at Macrogen Corporation (Seoul, Korea). The sequence reads were assembled by *de novo* genomic assembler, Velvet 1.2.10 (Zerbino and Birney 2008), and annotated by the Dual Organellar GenoMe Annotator (Wyman et al. 2004), ARAGORN v1.2.36 (Laslett and Canback 2004), and RNAmmer 1.2 Server (Lagesen et al. 2007). Using Geneious v8.1.6 (Biomatters Ltd., Auckland, New Zealand), the draft annotation was inspected and corrected manually using *Lactuca sativa* (DQ383816) as a reference.

The complete chloroplast genome (GenBank: MN334533) was 152,406 bp in length, consisting of large single-copy region (84,331 bp), small single-copy region (18,583 bp), and a pair of inverted repeats (24,746 bp). It contains 130 genes, including 87 protein-coding genes, six rRNA genes, and 37 tRNA genes. Based on whole cp genome sequences, the taxonomic position and evolutionary relationship of *S. leptocephalus* were determined based on 17 representatives of the tribe Cichorieae (Asteraceae) after alignment using MAFFT v.7 (Katoh and Standley 2013) (Figure 1). A maximum-likelihood tree produced by IQ-TREE (Nguyen et al. 2015) with 1000 replicate bootstrap (BS) analyses suggested that *S. leptocephalus* is sister to a clade containing other members of the alliance, i.e. *S. canariensis* and *S. acaulis*. Subtribes Lactucinae, Crepidinae, and Hyoseridinae were monophyletic, and within Hyoseridinae, the woody *Sonchus* alliance was sister to the clade containing weedy *Sonchus* species.

**Figure 1.**
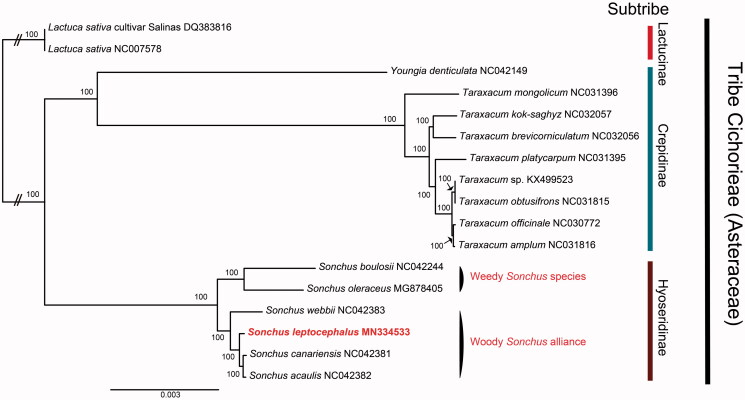
Maximum-likelihood tree of 17 species of the tribe Cichorieae (Asteraceae): two *Lactuca sativa* (DQ383816 and NC007578), six *Sonchus* (NC042382, NC042244, NC042381, MG878405, NC042383, and MN334533), eight *Taraxacum* (NC031816, NC032056, NC032057, NC031396, NC031815, NC030772, NC031395, and KX499523) and one *Youngia* (NC042149). The numbers above branches indicate bootstrap support values calculated with 1000 bootstrap replication test.
